# Retinal Detachment-Induced Müller Glial Cell Swelling Activates TRPV4 Ion Channels and Triggers Photoreceptor Death at Body Temperature

**DOI:** 10.1523/JNEUROSCI.0897-18.2018

**Published:** 2018-10-10

**Authors:** Hidetaka Matsumoto, Shouta Sugio, François Seghers, David Krizaj, Hideo Akiyama, Yasuki Ishizaki, Philippe Gailly, Koji Shibasaki

**Affiliations:** ^1^Departments of Ophthalmology,; ^2^Molecular and Cellular Neurobiology, Gunma University Graduate School of Medicine, Maebashi 371-8511, Japan,; ^3^Laboratory of Cell Physiology, Institute of Neuroscience, Université catholique de Louvain, B-1200 Brussels, Belgium, and; ^4^Department of Ophthalmology and Visual Sciences, Moran Eye Institute, University of Utah School of Medicine, Salt Lake City, Utah 84132

**Keywords:** glia, mechanical stimulus, retina, swelling, temperature, TRPV4

## Abstract

Using region-specific injection of hyaluronic acid, we developed a mouse model of acute retinal detachment (RD) to investigate molecular mechanisms of photoreceptor cell death triggered by RD. We focused on the transient receptor potential vanilloid 4 (TRPV4) ion channel, which functions as a thermosensor, osmosensor, and/or mechanosensor. After RD, the number of apoptotic photoreceptors was reduced by ∼50% in TRPV4KO mice relative to wild-type mice, indicating the possible involvement of TRPV4 activation in RD-induced photoreceptor cell death. Furthermore, TRPV4 expressed in Müller glial cells can be activated by mechanical stimuli caused by RD-induced swelling of these cells, resulting in release of the cytokine MCP-1, which is reported as a mediator of Müller glia-derived strong mediator for RD-induced photoreceptor death. We also found that the TRPV4 activation by the Müller glial swelling was potentiated by body temperature. Together, our results suggest that RD adversely impacts photoreceptor viability via TRPV4-dependent cytokine release from Müller glial cells and that TRPV4 is part of a novel molecular pathway that could exacerbate the effects of hypoxia on photoreceptor survival after RD.

**SIGNIFICANCE STATEMENT** Identification of the mechanisms of photoreceptor death in retinal detachment is required for establishment of therapeutic targets for preventing loss of visual acuity. In this study, we found that TRPV4 expressed in Müller glial cells can be activated by mechanical stimuli caused by RD-induced swelling of these cells, resulting in release of the cytokine MCP-1, which is reported as a mediator of Müller glia-derived strong mediator for RD-induced photoreceptor death. We also found that the TRPV4 activation by the Müller glial swelling was potentiated by body temperature. Hence, TRPV4 inhibition could suppress cell death in RD pathological conditions and suggests that TRPV4 in Müller glial cells might be a novel therapeutic target for preventing photoreceptor cell death after RD.

## Introduction

Retinal detachment (RD) causes photoreceptor cell death leading to visual decline, with ∼15,000 new patients of nontraumatic RD every year in the United States (Regillo and Bensen, 1988). RD is composed of various retinal disorders, including rhegmatogenous RD, age-related macular degeneration, and diabetic retinopathy. In most RD cases, visual acuity is decreased even after retinal reattachment, because of photoreceptor cell death that is evoked after RD. Therefore, identification of the mechanisms of photoreceptor cell death in the detached retina is required for establishment of therapeutic targets for preventing loss of visual acuity.

Pathological swelling of neurons and glia is an important feature of RD in mammals. Optical coherence tomography (OCT) obtains cross-sectional retinal images *in vivo* and has an advantage to examine the RD pathology in patients. In clinical settings, the OCT often demonstrates intraretinal edema in RD patients (Hagimura et al., 2000; Nakanishi et al., 2009). Moreover, in a primate model of RD, the cystoid degeneration can been observed in the inner retinal layers (Machemer, 1968; Machemer and Norton, 1969). In addition, many RD animal models revealed specific features of RD pathology in the inner retinal layers (Machemer, 1968; Machemer and Norton, 1969; Francke et al., 2005; Wurm et al., 2006). Morphological analysis in an animal model study revealed obvious Müller glial swelling after RD in the rabbit retina, pointing out the resemblance to human RD pathology (Francke et al., 2005). Furthermore, osmotic Müller glial cell swelling accompanied by a decrease in K^+^ conductance was observed in a porcine model of RD (Wurm et al., 2006). These reports suggest that the RD induces osmotic swelling of Müller glial cells by altering ion channel activity, but the molecular mechanisms have not been investigated.

The transient receptor potential vanilloid 4 (TRPV4) is a nonselective cation channel that was first described as an osmosensor capable of detecting hypotonic stimuli (Liedtke et al., 2000; Strotmann et al., 2000; Wissenbach et al., 2000; Nilius et al., 2001). We showed that TRPV4 mediates Müller glial osmosensation (Ryskamp et al., 2014; Lakk et al., 2017). TRPV4 can also be activated by heat (>27–34°C), the phorbol ester derivative 4α-phorbol 12,13 didecanoate, or lipids, including arachidonic acid metabolites (Güler et al., 2002; Watanabe et al., 2002a,b, 2003; Shibasaki et al., 2013). In addition, we found that TRPV4 was constitutively activated at physiological brain temperature to control neuronal excitability (Shibasaki et al., 2007b, 2015a,b; Hoshi et al., 2018).

Müller glial cells, which envelop photoreceptors, have pivotal functions: (1) cytokine-mediated protection of photoreceptor cells from death, (2) releasing antioxidant substances such as glutathione, and (3) buffering the elevated extracellular K^+^ and protect neuronal cells from glutamate and nitric oxide toxicity (Hertz, 2004). On the other hand, activated Müller glial cells cause cytotoxic effects in pathological retina. First, they express proinflammatory cytokines such as TNFα, IL1-β, and monocyte chemoattractant protein-1 (MCP-1; Murakami et al., 2013). Second, they produce free radicals and decrease glutamate uptake. Third, they lose extracellular K^+^ buffering, which leads to neuronal hyperactivation and excitotoxicity. In a previous study, we showed that the mechanosensing function of TRPV4 expressed in Müller glial cells can be activated by a swelling-induced membrane stretch and is important for maintaining cell volume (Ryskamp et al., 2014; Lakk et al., 2017). We, therefore, hypothesized that significant Müller glial swelling and photoreceptor degeneration in RD (Francke et al., 2005) may be linked by TRPV4 overactivation, possibly through the release of proinflammatory cytokines (Murakami et al., 2013).

A previous study showed elevated levels of the cytokine MCP-1 after RD, suggesting that Müller glial cells could release inflammatory cytokines that promote photoreceptor cell death through recruitment of macrophages in the RD sites (Nakazawa et al., 2007). However, it has not been revealed how the MCP-1 release in Müller glial cells is triggered by the RD pathogenesis. We expected that the Müller glial swelling and TRPV4 might be related to the MCP-1 release.

## Materials and Methods

### 

#### 

##### Animals.

All animal experiments followed guidelines in the ARVO Statement for the Use of Animals in Ophthalmic and Vision Research and were approved by the Gunma University Animal Care and Experimentation Committee. C57BL/6 mice were purchased from Japan SLC. TRPV4KO mice were previously generated by crossing heterozygous mice, and the genotypes were determined by PCR. Mice were fed standard laboratory chow and allowed *ad libitum* access to water in an air-conditioned room. All mice were used at 10 ± 2 postnatal weeks.

##### Generation of TRPV4-flox mice and Müller glia/astrocyte-specific conditional TRPV4KO mice.

Using AK7 mouse embryonic stem cells, we targeted the *Trpv4* gene by homologous recombination with a construct from the International KO Mice Consortium that bears the *loxP* site-flanked sixth exon (ID:79331; see Fig. 6*A*). After selection of several correctly targeted clones, we injected them into C57BL/6J mice host blastocysts. The embryos were transferred into pseudopregnant CD1 mice. In the offspring, chimeric males were selected on the basis of the coat color. They were mated with C57BL/6J females to assess the transmission of the targeted allele. Mice harboring the targeted allele were mated with *ROSA-Flp* females to have the selection cassette excised. The so obtained mice had the sixth exon of the *Trpv4* gene flanked with *loxP* sites (*Trpv4^lox^*). Homozygous mice for the *Trpv4lox* allele (*Trpv4^lox/lox^* mice) were crossed with a mouse line expressing the Cre recombinase under the *Pgk-1* promoter to verify the functionality of *loxP* sites. We obtained a constitutive *Trpv4* knock-out (KO) mouse line (*Trpv4*^−/−^). By using PCR on genomic tail DNA, we confirmed the functionality of the Cre-mediated recombination of the *lox*-flanked *Trpv4* exon. Forward (gctctggagaaagttcacac) and reverse (tgagatcccagtcctcatac) primers, targeting close upstream of the *Trpv4* sixth exon, allowed us to discriminate between wild-type (WT; 188 bp product), *loxP*-flanked (327 bp product), and knock-out (no product) alleles (see Fig. 6*B*).

To generate Müller glia/astrocyte-specific conditional TRPV4KO mice, the *Trpv4^lox/lox^* mice were crossed with hGFAP-Cre mice (Cai et al., 2007).

##### Induction of retinal detachment.

We modified a previously reported method for creating bullous and persistent RD (Matsumoto et al., 2013). Briefly, mice were anesthetized with an intraperitoneal injection of 50 mg/kg Nembutal, and the pupils were dilated with topical phenylephrine (5%) and tropicamide (0.5%). The temporal conjunctiva at the posterior limbus was incised and detached from the sclera. A 30 gauge needle (Dentronics) was used with the bevel pointed upward to create a sclerotomy 1 mm posterior to the limbus. A scleral tunnel was created, followed by scleral penetration into the choroid, which created a self-sealing scleral wound. A corneal puncture was made with a 30 gauge needle to lower the intraocular pressure. A 33 gauge needle connected to a 10 μl syringe (NanoFil 10-lL syringe, World Precision Instruments) was inserted into the subretinal space with the bevel pointed down. Then, 4 μl of 1% sodium hyaluronate (Healon, Abbott Laboratories Inc.) was injected gently, detaching ∼50% of the temporal–nasal neurosensory retina from the underlying retinal pigment epithelium (RPE). Finally, cyanoacrylate surgical glue (Webglue, Patterson Veterinary) was applied to the scleral wound, and the conjunctiva was reattached in the original position. Eyes with subretinal hemorrhage were excluded from analysis. In this RD model, photoreceptor cell death peaked ∼24 h after induction of RD (Matsumoto et al., 2014a,b, 2015).

##### TUNEL staining and visualization of macrophage and microglia.

Eyes with RD were enucleated and embedded in OCT compound (Tissue-Tek, Sakura Finetek Japan). Serial sections in the sagittal plane were cut into 10-μm-thick slices with a cryostat (CM3050 S, Leica) at −20°C and prepared for staining. We performed TUNEL assays according to the manufacturer's protocol (ApoTag Fluorescein In Situ Apoptosis Detection kit, Millipore). Finally, sections were counterstained with DAPI to visualize the nuclei. Cells that were TUNEL positive were counted in the outer nuclear layer (ONL) containing the photoreceptor cell bodies. The ONL area was also measured using ImageJ software (http://imagej.nih.gov/ij/; provided in the public domain by the National Institutes of Health, Bethesda, MD), and TUNEL-positive cell density in the ONL was calculated. Our previous experiments revealed that the center of the RD, which showed severe twist shapes, had significant increase of TUNEL-positive cells (Matsumoto et al., 2014b). We thus specifically chose the ONL regions ∼1000 μm from the injection site to exclude the center region. The average TUNEL-positive cell density in the two ONL regions per each retina was calculated as the representative TUNEL-positive photoreceptor cell density as the section amount (Matsumoto et al., 2014b). Then, the total average of the TUNEL-positive photoreceptor cell densities per ONL area was determined from three different sections. Microglia and migrated macrophages were visualized by anti-CD11b antibody (Abcam). The total average of the CD11b-positive cell densities per retina was determined as the TUNEL count. Photographs were taken with a fluorescence microscope (BX53, Olympus) equipped with a cooled CCD camera (DP80, Olympus) or a confocal microscope (LSM880, Zeiss Japan).

##### Immunohistochemistry.

Fixed retinal sections were washed with PBS and blocked in 3% bovine serum albumin in PBS containing 0.3% Triton X-100 (PBS-T). The sections were then incubated with anti-glutamine synthetase (rat monoclonal, 1:500, Abcam), anti-GFAP (rabbit polyclonal, 1:500, Dako), and anti-Ki67 (mouse monoclonal, 1:500; BD Biosciences) for 2 h at room temperature. After washing with PBS-T, primary antibodies were visualized using goat anti-rat IgG (Alexa488, 1:1000, Life Technologies Japan). Fluorescence images were captured using a fluorescence microscope (BX53, Olympus) equipped with a cooled CCD camera (DP80, Olympus) or a confocal microscope (LSM880, Zeiss Japan).

##### Quantitative real-time PCR assay.

Total RNA extraction and reverse transcription were performed as reported previously (Shibasaki et al., 2007a). After PCR, primers for MCP-1 were used as reported (Nakazawa et al., 2007): mMCP1 forward, 5′-ACTCACCTGCTGCTACTCATTCACC-3′; mMCP1 reverse, 5′-CTACAGCTTCTTTGGGACACCTGCT-3′. For relative comparison of each gene, we analyzed the Ct value of real-time PCR data with the ΔCt method normalizing by an endogenous control (β-actin RNA).

##### Acute dissociation of Müller glial cells.

Eyes were enucleated, and the retinas were carefully isolated from the eye balls and placed in cold Leibovitz 15 (L15) medium (Invitrogen) containing 11 mg/ml L15 powder, 20 mm d-glucose, 10 mm Na-HEPES, 2 mm Na-pyruvate, 0.3 mm Na-ascorbate, and 1 mm glutathione. To digest the extracellular matrix, retinae were incubated in L15 containing papain (7 U/ml, Worthington) for 1 h at room temperature. After digestion, retinae were rinsed, placed in cold L15 medium, and cut into 500 μm pieces. One or two of these pieces were triturated and plated on concanavalin A (1 mg/ml)-coated coverslips. One hour after plating, the cells were used for experiments. Müller glial cells were identified by their distinctive morphology. Under our experimental conditions, most plated cells maintained homeostasis for several hours at 25°C without substantial shifts in the baseline intracellular calcium concentration ([Ca^2+^]_i_) or the amplitudes of [Ca^2+^]_i_ responses to agonists.

##### Patch-clamp recording and calcium imaging.

Whole-cell patch-clamp recording was performed in a standard bath solution containing 140 mm NaCl, 5 mm KCl, 2 mm MgCl_2_, 2 mm CaCl_2_, 10 mm HEPES, and 10 mm glucose, pH7.4. Reversal potential was measured using voltage ramps (−100 to +100 mV in each 5 s interval). Pipette solutions for whole-cell recordings contained 120 mm K-gluconate, 20 mm KCl, 0.5 mm EGTA, 2 mm Mg-ATP, 2 mm K_2_-GTP, and 10 mm HEPES, pH7.4. Whole-cell recording data were sampled at 10 kHz and filtered at 5 kHz for analysis (Axon 200B amplifier with pCLAMP software, Molecular Devices). To apply stepped positive pressure to the acutely dissociated Müller glial cells, we used a high-speed pressure clamp (HSPC; HSPC-1, ALA). For *ex vivo* experiments, eye balls were dissected from mice and placed in ice-cold Krebs'–Ringer's solution containing (in mm) 119 NaCl, 2.5 KCl, 2.5 CaCl_2_, 1.3 MgSO_4_, 1.0 NaH_2_PO_4_, 26.2 NaHCO_3_, and 11 glucose, pH7.4, that had been saturated with 95% O_2_ and 5% CO_2_. The whole retinae were incubated with mitotracker dye to visualize the Müller glial cells, removed all vitreous by a medical quick absorber, and finally were explanted. The retinal whole-mounts were placed ganglion cell layer up. The endfeets of Müller glial cells were targeted for the whole-cell patch-clamp recordings at 37°C.

Fluo4 fluorescence was measured by Fluo-4 AM (Dojindo) in a standard bath solution. Fluo-4 AM was diluted to 2.5 μm in a standard solution with 0.1% pluronic F-127 (Life Technologies Japan) and loaded into the cells by incubating for 30 min at 37°C with 5% CO_2_. After incubation, excess Fluo-4 AM dye was washed out with three rinses of the standard solution. Fluorescence images of Fluo-4 AM were captured at 3 s intervals for 8 min with an upright fluorescence microscope (BX51WI, Olympus) equipped with a CMOS camera (Neo, Andor).

##### Müller cell culture.

Eyes from P6 mice were quickly removed and transferred to an ice-cold medium saturated with 95% O_2_ and 5% CO_2_. The medium (ACSF) contained (in mm) 119 NaCl, 2.5 KCl, 2.5 CaCl_2_, 1.3 MgSO_4_, 1.0 NaH_2_PO_4_, 26.2 NaHCO_3_, and 11 glucose. The eyes were cut to prepare the flat-mount shapes and embedded in 2% collagen gel. The retinal ganglion cell layers (100 μm thickness) were removed in the medium with a tissue slicer (VT-1200S, Leica). The retinae were digested using a papain dissociation system following the manufacturer's instructions [dissociation solution: DMEM/F-12 containing 16.5 U/ml activated papain (Worthington) and 124 U/ml DNase I (Sigma-Aldrich)] at 37°C for 15 min and maintained in a stationary culture in 10% serum-supplemented DMEM and F-12 (1:1). The removal of aggregates and cellular debris after 6–7 d yielded purified Müller glial cell cultures, which were maintained or passaged in DMEM/F-12 with 10% serum.

##### ELISA.

The eye tissues or cultured cells were put in TNE buffer [10 mm Tris-HCl, 150 mm NaCl, 1 mm EDTA, complete EDTA-free protease inhibitor mixture (Roche Applied Science), and 1 m Na_3_VO_4_]. Then, those were homogenized by Polytron homogenizer (PT6100, VWR) on ice. The protein amount was measured in an aliquot of homogenized samples using the Bio-Rad protein assay kit. The levels of MCP-1, IL-1β, and TNFα were determined with mouse MCP-1, IL-1β, and TNFα (R&D Systems) ELISA kits, according to the manufacturer's protocol. Briefly, the absorbance of the samples was measured by a spectrophotometer (MULTISKAN GO, Thermo Fisher Scientific) at 450 nm, and we calculated the release amount using Microsoft Excel.

##### Statistical analysis.

The Mann–Whitney *U* test was used to compare unpaired values of TUNEL-positive cell density and proinflammatory cytokine concentration. The data analyses were performed using Excel 2016 (Microsoft) with add-in software Statce14@. *p* < 0.05 was considered significant.

## Results

**Figure 1.**
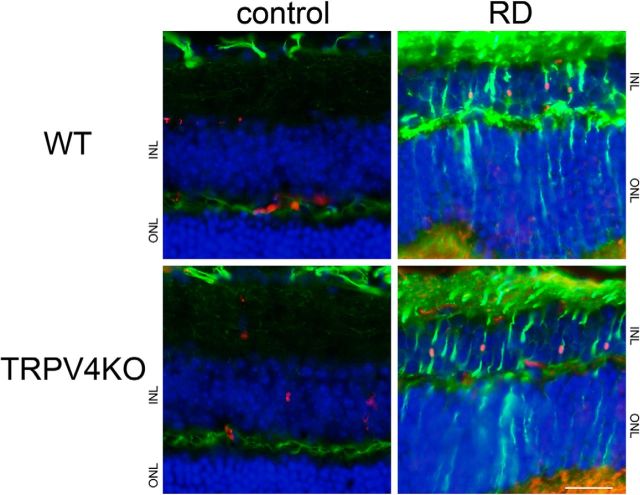
Our acute RD model evokes gliosis. Immunostaining of GFAP (a Müller gliosis marker, represented as green) and Ki67 (a proliferation marker, represented as red) was performed in RD retinae. Nuclei were stained by Hoechst (blue). The region of detachment from the RPE is represented as RD, in contrast to the normal region (represented as control). Scale bar, 100 μm. ONL, Outer nuclear layer; INL, inner nuclear layer.

**Figure 2.**
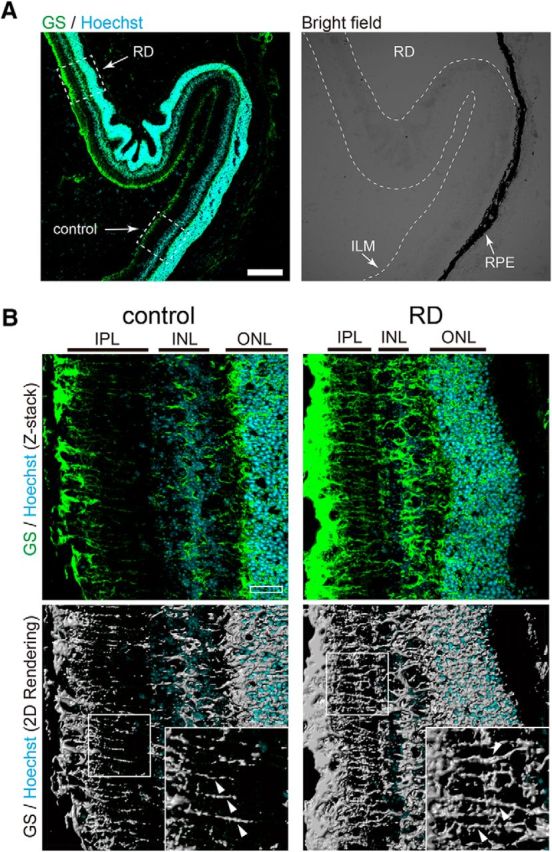
Müller glial cell swelling in an RD mouse model. ***A***, Immunostaining of glutamine synthetase (GS; a Müller glial cell marker, represented as green) was performed in RD retinae. Nuclei were stained by Hoechst (blue). In the bright-field image, the region of detachment from the RPE is represented as RD, in contrast to the normal region (represented as control). ILM, Inner limiting membrane. Scale bar, 200 μm. ***B***, High magnification of the region highlighted by a dashed box in ***A***. ONL, Outer nuclear layer; INL, inner nuclear layer; IPL, inner plexiform layer. Scale bar, 30 μm. Fluorescent images were converted to binary images by image software. Significant swelling of Müller processes in RD is indicated by arrowheads.

In this study, we developed a mouse model of RD induced by region-specific injection of hyaluronic acid. Previous findings indicate that RD induces the reactive gliosis of Müller glial cells (cell proliferation, cell hypertrophy, increased expression of intermediate filaments, decreased plasma membrane K^+^ conductance; Machemer, 1968; Machemer and Norton, 1969; Francke et al., 2005; Wurm et al., 2006). First, we examined whether the gliosis was observed in our RD model after 24 h of RD induction. Both RD retinae of WT and TRPV4KO mice showed upregulation of GFAP expression and an increase in proliferation compared with control retinae (Fig. 1). These results indicate that our acute RD model showed typical gliosis of Müller glial cells. None of the differences about the gliosis was observed between WT and TRPV4 retinae (Fig. 1). We focused on the significant swelling in Müller glia among the reactive gliosis (Machemer, 1968; Machemer and Norton, 1969), since we previously found that reactive gliosis of Müller glial cells takes place in our RD model (Matsumoto et al., 2014a). To assess Müller glial cell swelling in this acute RD model, we stained tissue sections with the anti-glutamine synthetase antibody to visualize Müller glial cells. Consistent with our previous observations, increased swelling of Müller glia was observed (Fig. 2). We performed the morphological analysis of Müller glial cells in 3-D graphical software (Fig. 2). Compared with control regions, the size of Müller glial cells in RD was increased by approximately threefold relative to cells in the control region (Fig. 2), indicating that this acute RD model is consistent with clinical characteristics of RD (Hagimura et al., 2000; Nakanishi et al., 2009). None of the differences about the swelling was observed between WT and TRPV4 retinae (data not shown).

**Figure 3.**
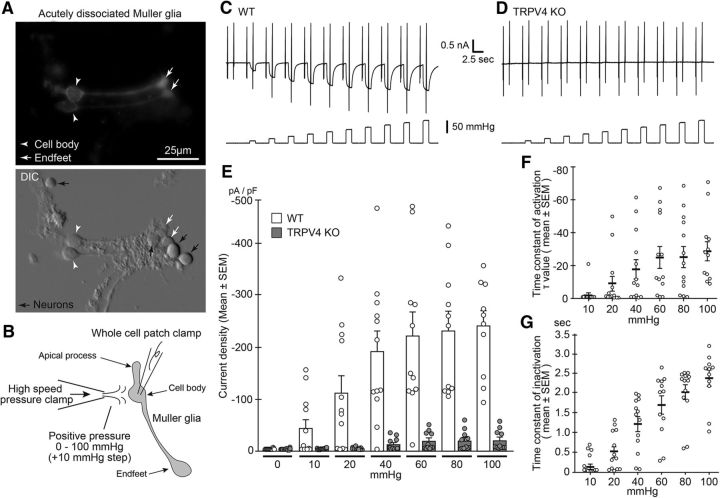
Müller glial TRPV4 is activated by mechanical stimuli. ***A***, Morphology of acutely dissociated Müller glial cells. White arrowheads and arrows represent soma and endfeet, respectively. Top, Immunostaining of acutely dissociated cells with glutamine synthetase (a Müller glial marker). Black arrows represent neurons. ***B***, Schematic drawing of a Müller glial cell and electrophysiological recording by stepped positive pressure stimulation (+10 mmHg per step). ***C***, ***D***, Representative traces of WT or TRPV4KO cells after application of stepped positive pressure with ramp pulses (−100 to +100 mV). The holding potential was at −60 mV. ***E***, Quantification of densities of positive pressure-evoked currents. WT, *n* = 10; TRPV4KO, *n* = 12. ***F***, Quantification of time constant of activation periods in WT recording (*n* = 10). ***G***, Quantification of time constant of inactivation periods in WT recording (*n* = 10).

We hypothesized that the RD-induced acute Müller glial swelling evoked a strong membrane stretch and might thus activate the TRPV4 channel. To examine it, we should perform whole-cell patch-clamp recording from the Müller glial cells in normal or RD retinal tissues. However, it was impossible to perform the experiments, since we had to remove the RPE to prepare the retinal slices even in normal retina. None of the preparation without removing RPE existed for retinal slice patch-clamp recording. Thus, it was entirely impossible to prepare control cells for the RD study. This specific background required unique experiments to prove the RD-induced Müller glial swelling evoked the TRPV4 activation. To explore whether Müller glial swelling induces a membrane stretch that could activate TRPV4 in the RD region, we prepared acutely isolated Müller glial cells (Fig. 3*A*). The morphology of the isolated Müller glial cells retained the characteristics that *in vivo*, and the cells could be easily distinguished based on their characteristic morphology (Fig. 3*A*). We next performed whole-cell patch-clamp recordings of RD Müller glial cells isolated from WT or TRPV4KO mice. To mimic the RD-induced membrane stretch, we used the HSPC method to apply stepped positive pressure (from 10 to 100 mmHg) to the cells (Fig. 3*B*). We observed a significant mechanical stimuli-evoked current in the WT cells, but not in the TRPV4KO cells, indicating that the positive pressure activated the mechanosensing activity of TRPV4 (Fig. 3*C–E*). Notably, the positive pressure did not activate any mechanosensing activity of TRPV4 in TRPV4-expressing HEK293 cells (data not shown). These results indicate that the Müller glia possess specific characteristics for the mechanosensing activity of TRPV4. The current densities increased with increasing pressure (Fig. 3*C–E*), and the time constant of activation and inactivation increased and decreased, respectively, depending on the amount of pressure applied only in WT cells (Fig. 3*F*,*G*).

**Figure 4.**
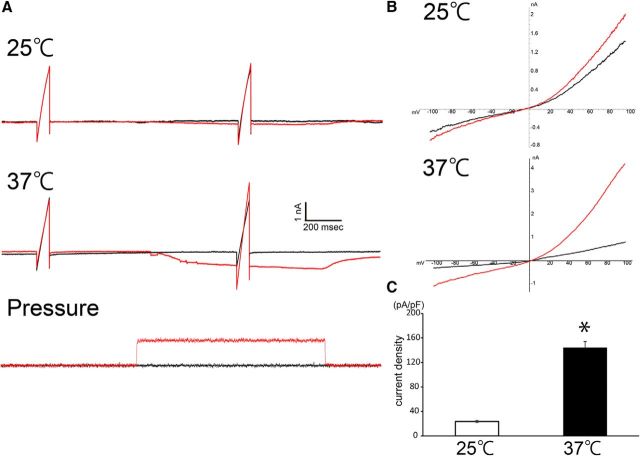
Müller glial TRPV4 is synergistically activated by mechanical stimuli and body temperature. ***A***, Representative traces at 25 or 37°C induced by application of minimal positive pressure (+10 mmHg) by the HSPC system (red traces) with ramp pulses (−100 to +100 mV) compared with basal traces (black traces). The holding potential was at −60 mV. ***B***, The outward rectified the current–voltage relationship of mechanical stimulus-evoked current (red trace) corresponding to the application of 10 mmHg pressure. The black trace represents the basal current–voltage relationship. ***C***, Quantification of densities of positive pressure-evoked currents (*n* = 5, *p = 0.00056, Student *t* test).

**Figure 5.**
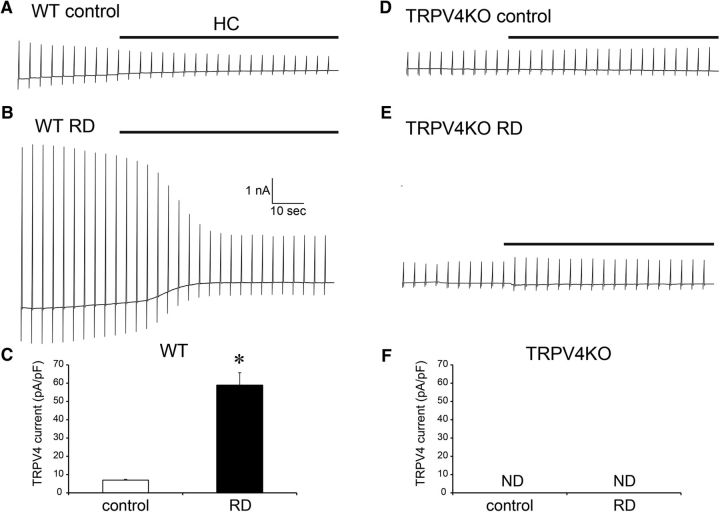
RD-induced Müller glial swelling activates TRPV4 at body temperature. ***A***, ***B***, Representative traces of Müller glia in a WT retinal explant at 37°C. The recording pipette was placed in the endfeet. The holding potential was at −60 mV. Ramp pulses (−100 to +100 mV) were applied in each 5 s interval. The black lines represent application of a TRPV4 antagonist HC (10 mm). Control is from normal retinal explants. RD is from RD-evoked retinal explants. ***C***, Quantification of densities of TRPV4-evoked currents in WT explants (*n* = 5, *p = 0.0058, Student *t* test). ***D***, ***E***, Representative traces of Müller glia in TRPV4KO a retinal explant at 37°C. The recording pipette was placed in the endfeet. The holding potential was at −60 mV. Ramp pulses (−100 to +100 mV) were applied in each 5 s interval. The black lines represent application of a TRPV4 antagonist HC (10 mm). Control is from normal retinal explants. RD is from RD-evoked retinal explants. ***F***, Quantification of densities of TRPV4-evoked currents in TRPV4KO explants (*n* = 5). We failed to observe any TRPV4 currents.

Based on our previous findings that (1) TRPV4 is activated in neural cells at temperatures >34°C (Shibasaki et al., 2007b), including the Müller glial cells (Lakk et al., 2017); (2) various ligands combined with increased temperature had synergistic potentiation effects on TRVP4 activation (Gao et al., 2003); and (3) TRPV4 is constitutively active at physiological body temperature (Shibasaki et al., 2015b), we expected that body temperature significantly reduced the TRPV4-activation threshold against the mechanical stimuli. Hence, we performed whole-cell patch-clamp recordings of Müller glial cells using the HSPC method at a physiological temperature. We applied a minimal membrane stretch (10 mmHg) to acutely dissociated Müller glial cells at 25 or 37°C and recorded the mechanical stimuli-evoked TRPV4 currents at each temperature (Fig. 4). Compared with 25°C conditions, the physiological temperature (37°C) significantly potentiated TRPV4 currents (Fig. 4). Notably, not all the cells changed the morphology by the application of the minimal membrane stretch (10 mmHg). Thus, it is highly possible that the marked cell swelling of Müller glial cells in RD (approximately three times increase in Fig. 2) activates TRPV4 and accelerates the death of photoreceptors in RD. Especially, the body temperature condition significantly reduced the TRPV4-activation threshold against the mechanical stimuli and was important to activate TRPV4 by the membrane stretch in Müller glial cells (Fig. 4). Next, we performed *ex vivo* whole-cell patch-clamp recording. In this experiment, retinae were incubated with mitotracker dye to visualize the Müller glial cells, removed all vitreous, and finally were explanted. The retinal whole-mounts were placed ganglion cell layer up. We recorded from the endfeets of Müller glia at 37°C. In WT control retinae, small inward currents were observed in the beginning of recordings, and these were inhibited by HC067047 (HC), a specific TRPV4 antagonist (Fig. 5*A*,*C*). In contrast to these results, none of the inward currents were observed in TRPV4KO control retinae, and the HC did not have any inhibiting effects (Fig. 5*D*,*F*). These results indicate that Müller glial TRPV4 is weakly activated in our whole-mount preparations. In contrast to WT control retinae, large inward currents were observed in WT RD retinae, and these were effectively inhibited by the HC application (Fig. 5*B*,*C*). None of the inward currents were observed in TRPV4KO RD retinae, and the HC did not have any inhibiting effects (Fig. 5*E*,*F*). Together, these results indicate that RD-induced Müller glial swelling induces membrane stretch that could activate TRPV4.

**Figure 6.**
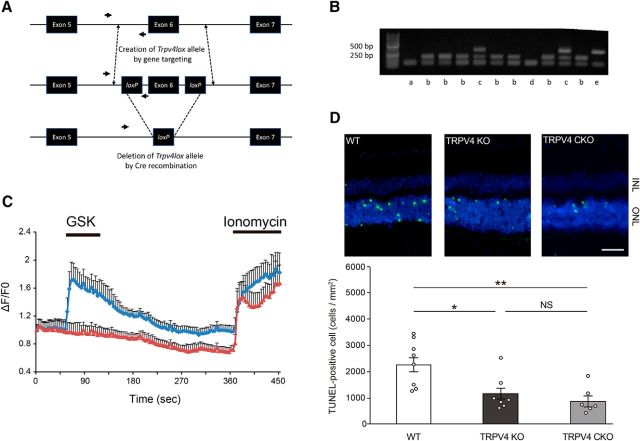
Müller glial TRPV4 activation accelerates photoreceptor cell death in RD. ***A***, Genetic manipulation of the *Trpv4* gene allowing conditional deletion of its sixth allele. The top row shows the wild-type locus. Arrows show the sequence targeted by the primers for the genotyping PCR. The middle row shows the targeted *Trpv4* gene, with the exon 6 flanked by *loxP* recombination sites. The bottom row shows the disrupted gene after Cre recombination. ***B***, PCR genotyping of blank (a; showing a nonspecific band), *Trpv4*^w/w^ (b; 188 bp fragment), *Trpv4*^wt/lox^ (c; 188 and 327 bp fragments), *Trpv4*^−/−^ (d; KO allele where the reverse primer site is not present), and Trpv4^lox/lox^ (e; 327 bp fragment). ***C***, Müller glia/astrocyte-specific TRPV4 conditional KO mice (TRPV4CKO) were generated by crossing hGFAP-Cre mice with TRPV4-flox mice. Shown are representative traces of [Ca^2 +^]_i_ changes in cultured Müller glial cells (blue diamond line, WT, *n* = 58 cells; red triangle line,TRPV4CKO, *n* = 46 cells). Four days after culture, [Ca^2+^]_i_ changes were measured by Fluo-4 AM. The data were quantified as Δ*F*/*F*0. A TRPV4 agonist (10 nm GSK) was applied during recording. At the end of each experiment, we applied ionomycin (5 μm) to identify the surviving cells. ***D***, Representative images of TUNEL staining in RD retinal tissues (WT, TRPV4KO, and TRPV4CKO). Conventional TRPV4KO mice showed significantly less photoreceptor cell death than WT mice 24 h after retinal detachments (*p = 0.06706, *n* = 7 or 8 eyes). The TRPV4CKO mice showed significantly less photoreceptor cell death than WT mice (**p = 0.020863, *n* = 6 eyes). The TRPV4CKO results were perfectly matched with those in TRPV4KO, indicating that Müller glial TRPV4 activation accelerates photoreceptor cell death in RD. ONL, Outer nuclear layer; INL, inner nuclear layer.

Next, we used TUNEL staining to examine photoreceptor cell death in WT or TRPV4KO mice with RD. Since *Trpv4* was deleted in all cell types in TRPV4KO mice, we could not conclude the Müller glial involvement only from this mouse model. Hence, we generated Müller glia/astrocyte-specific conditional TRPV4KO mice (TRPV4CKO) by crossing TRPV4-flox mice (Fig. 6*A*,*B*) with hGFAP-Cre mice. We confirmed the functional loss of *Trpv4* in Müller glial cells by Ca^2+^ imaging experiments (Fig. 6*C*). The number of dead photoreceptors in TRPV4KO mice was 50% less than that for WT mice (Fig. 6*D*). We previously reported that retinal expression of TRPV4 is confined to Müller cells, subtypes of retinal ganglion cells and microvascular endothelial cells, but that photoreceptors do not express TRPV4 (Ryskamp et al., 2011; Jo et al., 2015; Phuong et al., 2017). Consistent with those previous reports and our data (Figs. 1–5), the number of dead photoreceptors in TRPV4CKO mice was 50% less than that for WT mice, and the results were perfectly matched with the result in the conventional TRPV4KO (Fig. 6*D*). Together, these results indicate that Müller glial TRPV4 activation in RD was associated with the induction of photoreceptor cell death.

**Figure 7.**
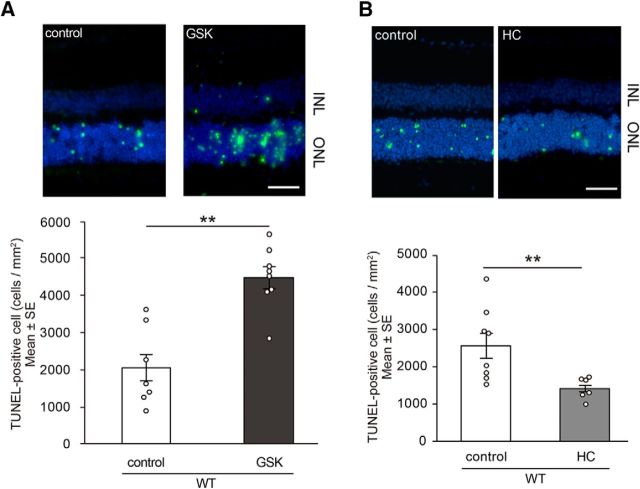
***A***, TRPV4 agonist or control PBS was injected into the subretinal space when generating retinal detachment. The eyes with TRPV4 agonist (GSK, 1 mm) showed a significant increase in photoreceptor cell death compared with control eyes 24 h after retinal detachments (**p = 0.002622, *n* = 7). Shown are representative images of TUNEL staining in RD retinal tissues (control or TRPV4 agonist). The graphs show mean ± SEM. Scale bar, 50 μm. ***B***, TRPV4 inhibitor (*n* = 8) or control PBS (*n* = 6) was injected into the subretinal space when creating retinal detachment for WT mice. The eyes with TRPV4 inhibitor (HC, 1 mm) showed significantly less photoreceptor cell death compared with control eyes 24 h after retinal detachments (**p = 0.009823). Shown are representative images of TUNEL staining in RD tissues from WT mice (control or TRPV4 inhibitor). The graphs show mean ± SEM. Scale bar, 50 μm. ONL, Outer nuclear layer; INL, inner nuclear layer.

To confirm TRPV4 involvement in RD, we injected GSK1016790A (GSK), a potent TRPV4 agonist (Shibasaki et al., 2015b), into WT mouse eyes simultaneously with hyaluronic acid used to generate acute RD. The subretinal injection of GSK significantly increased the number of dead photoreceptors compared with that of control retinas (Fig. 7*A*), suggesting that Müller glial TRPV4 activation significantly increases photoreceptor cell death in RD. In contrast, injection of the TRPV4 antagonist HC into the retina after generation of acute RD in mice resulted in a significant reduction in the number of TUNEL-positive cells compared with untreated (control) mice (Fig. 7*B*). Hence, TRPV4 inhibitors could be a therapeutic tool to prevent disease progression after RD.

**Figure 8.**
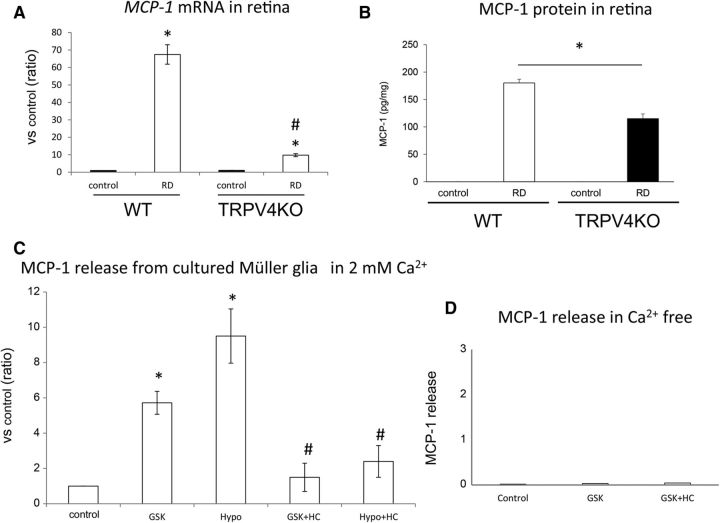
TRPV4 activation triggers MCP-1 release from Müller glial cells and leads to photoreceptor apoptosis in the detached retina. ***A***, Real-time PCR assays were performed using specific MCP-1 primers. The values were normalized against β-actin expression, and the bar graphs compare values with the MCP-1/β-actin expression level of control eyes in WT mice. Asterisks (vs control) and hash tags (vs WT RD) represent a significant difference at *p* < 0.01 (*p = 0.000116, ^#^*p* = 0.000232, Student *t* test, *n* = 5). ***B***, ELISAs of MCP-1 protein were performed in control and RD eyes for WT and TRPV4KO mice. The asterisk represents a significant difference at *p* < 0.05 (*p* = 0.0010509, Mann–Whitney *U* test, *n* = 6). ***C***, ELISA assays of MCP-1 protein performed using cultured Müller glial cells from WT mice. Asterisks (vs control) and hash tags (vs GSK or Hypo) represent a significant difference at *p* < 0.01 (*GSK, *p* = 0.000247; *Hypo, *p* = 0.00944; ^#^GSK+HC, *p* = 0.001683; ^#^Hypo+HC, *p* = 0.00092; *t* test, *n* = 4). ***D***, ELISAs of MCP-1 protein were performed using the supernatant of cultured Müller glial cells (from WT mice) under Ca^2+^-free conditions. Only the small amount of MCP-1 release was observed independent from TRPV4 activation or inhibition (GSK or HC). These results were perfectly different from the physiological Ca^2+^ condition (2 mm) shown in Figure 5*C*. Thus, we can conclude that TRPV4-triggered Ca^2+^ influx evokes the MCP-1 release from Müller glial cells.

**Figure 9.**
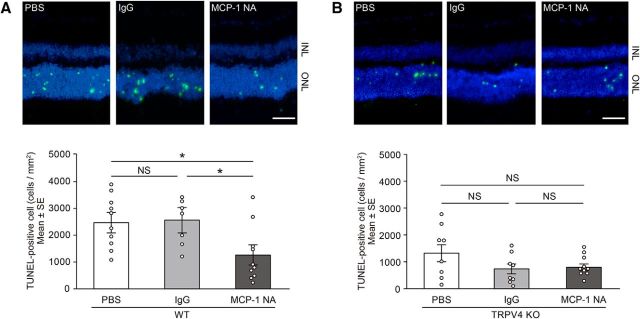
TRPV4 activation occurs upstream of MCP-1 release in Müller glial cells and accelerates photoreceptor cell death in RD. Control anti-IgG antibody, neutralizing anti-MCP-1 antibody, or control PBS was injected into the subretinal space when creating retinal detachment for WT and TRPV4KO mice. ***A***, Representative images of TUNEL staining in RD retinal tissues from WT mice (PBS, IgG, or neutralizing anti-MCP-1 antibody). The WT eyes with neutralizing anti-MCP-1 antibody (100 μg/ml) showed significantly less photoreceptor cell death compared with control eyes (PBS or IgG) 24 h after retinal detachments (*p = 0.012611 vs PBS, *p = 0.012851 vs IgG; *n* = 10). ***B***, Representative images of TUNEL staining in RD retinal tissues from TRPV4KO mice (PBS, IgG, or neutralizing anti-MCP-1 antibody). The TRPV4KO eyes (*n* = 8) with neutralizing anti-MCP-1 antibody (100 μg/ml) did not show a significant difference in photoreceptor cell death compared with control eyes 24 h after retinal detachments. ONL, Outer nuclear layer; INL, inner nuclear layer.

**Figure 10.**
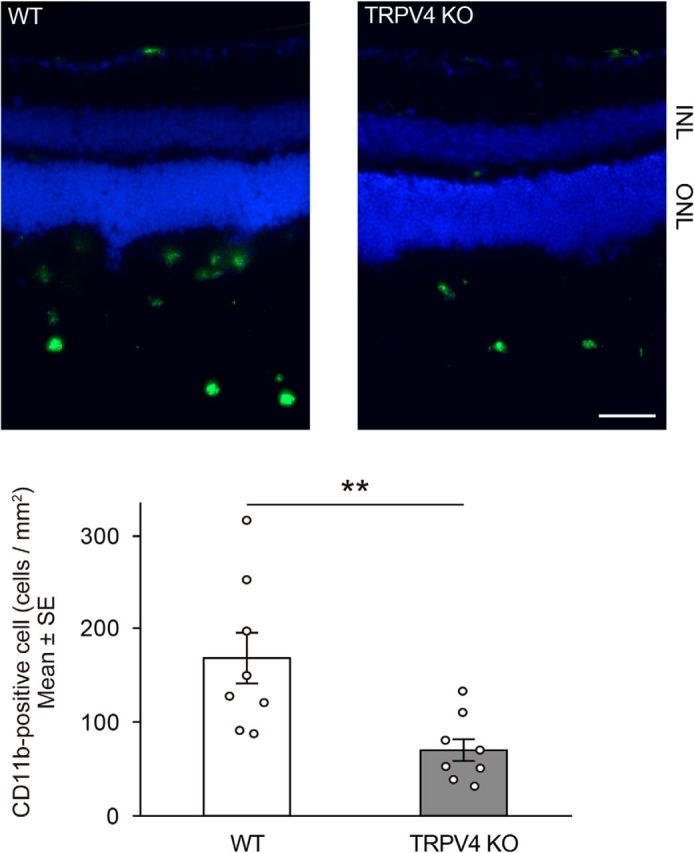
RD-induced TRPV4 activation recruits macrophages in subretinal space. Immunostaining of CD11b (a macrophage marker, represented as green) was performed in RD retinae. Nuclei were stained by Hoechst (blue). Shown is quantification of CD11b-positive cell numbers in WT or TRPV4 retinae 24 h after retinal detachments (*n* = 8, **p = 0.00632, Student *t* test). Scale bar, 100 μm. ONL, Outer nuclear layer; INL, inner nuclear layer.

**Figure 11.**
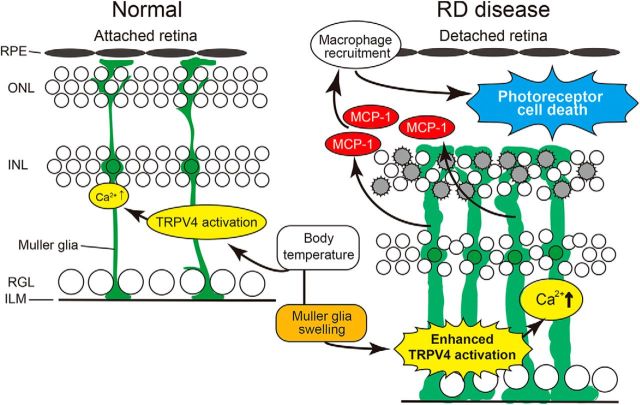
RD pathological condition shifts the Müller glial TRPV4 activation and accelerates photoreceptor cell death. The green color represents the morphology of the Müller glial cells in normal or RD retinas. In normal retina, TRPV4 is activated by body temperature and contributed to the homeostatic functions of Müller glial cells. In contrast, RD induced significant Müller glial swelling, and this caused further activation of TRPV4 by membrane stretch in addition to that by body temperature. TRPV4 activation induced the Ca^2+^ influx and evoked MCP-1 release from the Müller glial cells. The MCP-1 recruited many macrophages, and those cells attacked and killed photoreceptors (shown by gray color). Thus, RD adversely impacts photoreceptor viability via TRPV4-dependent MCP-1 release from Müller glial cells.

We next characterized the molecular mechanisms by which TRPV4 activation in Müller glial TRPV4 promotes photoreceptor cell death. Many previous reports indicated that Müller glial cells release cytokines that can be toxic to photoreceptors (Nakazawa et al., 2007, 2011). Furthermore, TRPV4 activation was shown to be associated with cytokine release in nonretinal tissues (Ye et al., 2012). Here we focused on release of MCP-1, a Müller glial cytokine known to induce death of photoreceptors through the recruitment of macrophages (Nakazawa et al., 2007). We hypothesized that TRPV4 activation might occur upstream of MCP-1 release in Müller glial cells under RD conditions. We first examined expression levels of *MCP-1* mRNA in WT or TRPV4 retinae under normal (control) and RD conditions. Consistent with findings by Nakazawa et al. (2007), RD significantly induced *MCP-1* mRNA expression and increased MCP-1 protein levels in WT retinae, but these changes were not observed for TRPV4KO retinae (Fig. 8*A*,*B*). Together with the previous report that clearly demonstrated that MCP-1 release is caused by Müller glial cells (Nakazawa et al., 2007), our results suggest that Müller glial TRPV4 activation induced by RD might trigger MCP-1 release. To confirm this possibility, we prepared cultured Müller glial cells and quantified MCP-1 release induced by TRPV4 activation or inhibition (Fig. 8*C*). Application of GSK significantly increased MCP-1 release compared with control conditions (Fig. 8*C*). Interestingly, hypotonic stimuli (200 mOsm/kg) further increased MCP-1 release compared with control conditions or GSK application (Fig. 8*C*). Since hypotonic stimuli evoked significant cell swelling, the swelling-induced TRPV4 activation might significantly affect the TRPV4 activation. Both the GSK and hypotonic effects were abolished by application of the selective TRVP4 inhibitor HC (Fig. 8*C*). Furthermore, we examined the release of other cytokines such as TNFα or IL-1β, whose involvement in RD-induced photoreceptor cell death was reported. TNFα or IL-1β was not released from the Müller glial cells after the TRPV4 activation (data not shown). These results indicate that MCP-1 release was specifically triggered by the TRPV4 activation. We expected that TRPV4-induced Ca^2+^ influx was required for the MCP-1 release. Hence, we examined the MCP-1 release in Ca^2+^-free conditions. None of the MCP-1 release was observed in the Ca^2+^-free conditions after TRPV4 activation (Fig. 8*D*). These results demonstrate that TRPV4-induced Ca^2+^ influx and intracellular Ca^2+^ signaling evoke the MCP-1 release from mechanically stressed Müller glia. We hypothesize that this cytokine might be a critical death signal from Müller glial cells to photoreceptors after RD. To further examine the involvement of TRPV4 in MCP1 release induced by RD, we injected neutralized anti-MCP-1 antibody simultaneously with hyaluronic acid used to generate acute RD. Consistent with the previous report (Nakazawa et al., 2007), the neutralized antibody significantly suppressed cell death of photoreceptors in WT mice, although control IgG did not have any effect (Fig. 9*A*). However, in TRPV4KO mice the neutralized antibody did not suppress the cell death (Fig. 9*B*), indicating that Müller glial cell swelling activates TRPV4 and triggers MCP-1 release (Figs. 1–8). It was previously reported that MCP-1 released from the Müller glial cells induced recruitment of macrophages (Nakazawa et al., 2007), and the cells attack photoreceptors and promote cell death. Hence, we examined the macrophage distributions in subretinal space under our RD model (Fig. 10). In WT mice, many macrophages were recruited in subretinal space (Fig. 10). In contrast, the cell numbers were significantly reduced (approximately one-third vs WT) in TRPV4KO mice (Fig. 10). Thus, we propose that the RD adversely impacts the viability of photoreceptors via TRPV4-dependent cytokine release (Fig. 11), suggesting that injection of a TRPV4 inhibitor after RD can block photoreceptor cell death by reducing MCP-1 release.

## Discussion

In this study, we focused on Müller glial cell swelling and membrane stretch activation of TRPV4 in RD pathophysiological conditions. We found that RD-induced Müller glial cell swelling significantly activated TRPV4 channels (Figs. 2–5).

We revealed for the first time that body temperature significantly reduced the TRPV4-activation threshold against the mechanical stimuli (Fig. 4), indicating that body temperature significantly elevated TRPV4 sensitivity to membrane stretch as a result of a synergistic effect of temperature and mechanical stimuli. Indeed, our *ex vivo* electrophysiological recordings of Müller cells revealed membrane stretch-dependent TRPV4 activation at body temperature in the RD retinae (Fig. 5). In another study, we revealed that the synergistic effects of temperature and mechanical stimuli in TRPV4 expressed by Müller glial cells are specifically required to regulate synaptic transmission in normal retina (unpublished results). We also previously reported that body temperature constitutively activates neural TRPV4 and regulates brain activities (Shibasaki et al., 2007b, 2015b; Shibasaki, 2016). Therefore, we hypothesize that our observations concerning the contribution of Müller glial TRPV4 to synaptic transmission by retinal neurons through effects of body temperature might apply to homeothermic animals in general (Hanaoka et al., 2013). Compared with cold-blooded animals, birds and mammals must perform many complex behaviors that require a finely tuned visual transmission system. Thus, homeostatic constitutive TRPV4 activation by body temperature could contribute to this visual fine-tuning by taking advantage of a constant body temperature. On the other hand, temperature-dependent TRPV4 activation can negatively affect membrane stretch-dependent activation of TRPV4 in the presence of pathological cell swelling (Figs. 2–5), which is supported by our finding of a synergistic-potentiated activation of TRPV4 by body temperature and membrane stretch (Figs. 4, 5).

RD was previously shown to induce MCP-1 release from Müller glial cells, and MCP-1 was shown to promote photoreceptor cell death through the recruitment of macrophages (Nakazawa et al., 2007). The molecular mechanism by which MCP-1 release occurs after RD was previously unknown. Given that Müller glial cells perform key housekeeping, osmoregulatory, and mechanosensory functions in the retina (Ryskamp et al., 2014), we hypothesized that excessive cell swelling might modulate calcium-dependent release of proinflammatory metabolites by activating the resident TRPV4 channels. In this study, we revealed that swelling-induced TRPV4 activation triggered Ca^2+^ influx and evoked MCP-1 release in Müller glial cells (Figs. 1–5, 8), consistent with previous studies showing that glial TRPV4 activation triggered cytokine release (Shi et al., 2013). Indeed, our Müller glia-specific conditional KO mice can significantly reduce the RD-induced photoreceptor cell death (Fig. 6), and the reduction ratio of dead photoreceptors was perfectly the same as the conventional TRPV4KO mice (Fig. 6). These results indicate that RD-induced Müller glial swelling triggers TRPV4 activation and evokes the MCP-1 release from the Müller glia (Fig. 11). Furthermore, TRPV4 was also found to be involved in MCP-1 expression in brown adipose tissue (Ye et al., 2012). Notably, in this study we showed that TRPV4-induced Ca^2+^ influx by cell swelling occurs upstream of MCP-1 release from Müller glial cells (Fig. 8). This finding is consistent with our other previous studies showing the involvement of TRP-channel mechanosensor functions in a variety of physiological functions, including the following: (1) TRPV2-dependent sensation of membrane stretch by developing neurons, which enhances axon outgrowth (Shibasaki et al., 2010; Sugio et al., 2017); (2) TRPV2 mechanosensitivity that regulates intestinal motility (Regillo and Bensen, 1988); (3) body temperature- and movement-dependent activation of choroid plexus epithelial cells that further activates TRPV4 through epoxyeicosatrienoic acid production (Takayama et al., 2014); and (4) TRPV4-dependent sensation of urinary bladder distension, which can be converted to ATP signals in the micturition reflex pathway (Mochizuki et al., 2009; Shibasaki, 2016). The mechanisms by which TRPV4 contributes to Müller glial MCP-1 release remain to be clarified. It is reported that increase in the intracellular Ca^2+^ level activated inflammasome (Murakami et al., 2012), and caused the protein processing from pro IL-1β to IL-1β (Xiao et al., 2013). It is possible that TRPV4-induced Ca^2+^ influx activates the inflammasome signaling pathway, and induces MCP-1 release from the Müller glial cells.

Hence, TRPV4 inhibition could suppress cell death in RD pathological conditions (Fig. 7) and suggests that TRPV4 in Müller glial cells might be a novel therapeutic target for preventing photoreceptor cell death after RD (Fig. 11). Injection of a TRPV4 blocker into the vitreous or anterior chamber of eyes affected by rhegmatogenous RD might prevent photoreceptor cell death in advance of retinal reattachment surgery. Moreover, a TRPV4 blocker could be added to infusions made during vitrectomy that could also prevent photoreceptor cell death. For eyes with serous RD, such as central serous chorioretinopathy and age-related macular degeneration, intravitreal injection of a TRPV4 blocker could delay photoreceptor cell death until successful retinal reattachment by laser photocoagulation, photodynamic therapy, intravitreal injection of antivascular endothelial growth factor, or other treatments can be performed.

The role of TRPV4 in other retinal diseases has not been explored. In diabetic retinopathy, Müller glial cells are swollen, resulting in diabetic macular edema (Fine and Brucker, 1981). Levels of extracellular MCP-1 have been shown to be significantly elevated in patients with diabetic macular edema, and levels of MCP-1 could be significantly reduced after treatment, in association with a reduction in overall central macular thickness (Owen and Hartnett, 2013). Furthermore, Müller cells are also swollen in retinal vein occlusion (Rehak et al., 2009). Vitreous fluid MCP-1 levels are reportedly correlated with retinal vascular permeability and the severity of macular edema (Noma et al., 2013). Therefore, TRPV4 in Müller glial cells might be related to the pathogenesis of these disorders. In many RD models, reactive gliosis was observed (Machemer, 1968; Machemer and Norton, 1969; Francke et al., 2005; Wurm et al., 2006). Especially, Müller glial cell swelling was a significant observation and indicates that the swelling resembles human RD pathology (Francke et al., 2005). We found that the swelling-induced membrane stretch was enough to activate TRPV4 at body temperature (Figs. 3–5) and can cause MCP-1 release (Fig. 8). It was reported that the released MCP-1 recruited the macrophages near photoreceptors and the cells killed the photoreceptors (Nakazawa et al., 2007). Thus, the TRPV4 and MCP-1-induced macrophage recruitment (Figs. 10, 11) might be related to the disease progression of diabetic retinopathy or retinal vein occlusion.

The mechanisms by which TRPV4 contributes to Müller glial cell swelling remain to be clarified. TRPV4 is a nonselective cation channel through which Ca^2+^ enters the cell from the extracellular space in both neurons and glia (Ryskamp et al., 2014; Shibasaki, 2016; Lakk et al., 2017). Because the elevation in intracellular Ca^2+^ levels is a critical factor in brain edema (Hoshi et al., 2018), Ca^2+^ influx through TRPV4 may directly contribute to Müller glial cell swelling. Furthermore, TRPV4 activation was shown to induce water transport through Ano1, a Ca^2+^-activated Cl^−^ channel (Takayama et al., 2014) and is primed by water influx via AQP4 (Jo et al., 2015). Therefore, RD-induced TRPV4 activation could cause Ca^2+^ influx and subsequent water transport through AQP4 and Ca^2+^-activated channels. A nonmutually exclusive possibility is that TRPV4-mediated influx of other cations contributes to cell swelling. For example, neuronal swelling is partly caused by prolonged increases in intracellular Na^+^ that result in osmotic imbalances and water entry (Jo et al., 2016). Additionally, TRPV4 could promote swelling by serving as an osmotic sensor that mediates changes in osmotic pressure (Nakanishi et al., 2009; Toft-Bertelsen et al., 2017) or through activation induced by membrane stretch (Mochizuki et al., 2009; Shibasaki, 2016). Therefore, the cell swelling itself could recurrently activate TRPV4 to exacerbate effects of RD. Future studies will reveal the molecular mechanisms about the interaction between TRPV4 and swelling.
